# Supplementary material for: Complexome profiling of the Chlamydomonas psb28 mutant reveals TEF5 as an early PSII assembly factor
Source: Plant Cell. 2025 Mar 17;37(6):koaf055. doi: 10.1093/plcell/koaf055 (PMC12164590; doi:10.1093/plcell/koaf055)
Supplement: koaf055_Supplementary_Data [file koaf055_supplementary_data.zip › psb28_interactive.zip!/plots/intensity/qualityPlots/peptides/TEF9_Cre12.g519300.t1.2.html]

### Cre12.g519300.t1.2

Trivial name: TEF9  
SeqCoverage [median]: 22.10  
Mass: 27.45 kDa  
  
 

| Sequence | MissedCleavage | Mass [Da] | wtIntensity | muIntensity | Score |
| --- | --- | --- | --- | --- | --- |
| ALPAAGPDGVRPPSPNDSVIASK | 1 | 2215.16 | 39170.78 | 93379.74 | 134.86 |
| ALPAAGPDGVRPPSPNDSVIASKENER | 2 | 2743.39 | 0.00 | 6684.79 | 67.83 |
| ELGDEIR | 0 | 830.41 | 21351.15 | 27155.16 | 120.52 |
| ELGDEIRDLK | 1 | 1186.62 | 0.00 | 839.82 | 38.57 |
| ELGDEIRDLKK | 2 | 1314.71 | 0.00 | 2766.92 | 121.08 |
| KALPAAGPDGVRPPSPNDSVIASK | 2 | 2343.25 | 0.00 | 633.83 | 48.87 |
| KELLKGEYR | 2 | 1134.64 | 0.00 | 851.80 | 57.17 |
| TRELGDEIR | 1 | 1087.56 | 0.00 | 965.86 | 49.35 |
| VFQFTSWP | 0 | 1010.49 | 452.24 | 0.00 | 86.11 |
|  |  |  | 60974.2 | 133277.9 |  |
